# Targeting the Kynureninase–HDAC6–Complement Axis as a Novel Therapeutic Strategy in Glioblastoma

**DOI:** 10.3390/epigenomes9030027

**Published:** 2025-07-28

**Authors:** Arif Ul Hasan, Sachiko Sato, Mami Obara, Yukiko Kondo, Eiichi Taira

**Affiliations:** Department of Pharmacology, School of Medicine, Iwate Medical University, Yahaba 028-3694, Japan

**Keywords:** BET inhibitor, complement system, epigenetic therapy, glioblastoma, HDAC6, KYNU, kynurenine pathway, TCGA

## Abstract

**Background/Objectives:** Glioblastoma (GBM) is an aggressive brain tumor known for its profound heterogeneity and treatment resistance. Dysregulated complement signaling and epigenetic alterations have been implicated in GBM progression. This study identifies kynureninase (KYNU), a key enzyme in the kynurenine pathway, as a novel regulator of complement components and investigates its interaction with histone deacetylase 6 (HDAC6) in the context of therapeutic targeting. **Methods:** KYNU expression, and its association with complement signaling in GBM, were analyzed using publicly available datasets (TCGA, GTEx, HPA). Pathway enrichment was performed via LinkedOmics. In vitro studies in GBM cell lines (U87, U251, T98G) assessed the effects of KYNU silencing and treatment with an HDAC6 inhibitor (tubastatin) and a BET inhibitor (apabetalone) on gene expression and cell viability. **Results:** Bioinformatic analyses revealed significant overexpression of KYNU in GBM tissues compared to normal brain tissue. KYNU expression was positively associated with genes involved in complement and coagulation cascades. In vitro experiments demonstrated that KYNU silencing reduced the expression of C3, C3AR1, and C5AR1 and suppressed GBM cell viability. Treatment with tubastatin, while reducing viability, paradoxically upregulated complement genes, suggesting potential limitations in therapeutic efficacy. However, this effect was mitigated by KYNU knockdown. Combined treatment with apabetalone and tubastatin effectively suppressed KYNU expression and enhanced cytotoxicity, particularly in cells with high complement expression. **Conclusions:** Our findings establish the KYNU–HDAC6–complement axis as a critical regulatory pathway in GBM. Targeting KYNU-mediated complement activation through combined epigenetic approaches—such as HDAC6 and BET inhibition—represents a promising strategy to overcome complement-driven resistance in GBM therapy.

## 1. Introduction

Gliomas are primary brain tumors that typically arise from genetically altered neural stem or progenitor cells with tumor-initiating potential. Based on their microscopic features and molecular characteristics, gliomas are classified by the World Health Organization (WHO) into central nervous system (CNS) grades 1–4, representing increasing levels of malignancy [1]. Glioblastoma, *IDH*-wild-type CNS WHO grade 4—previously known as glioblastoma multiforme (GBM) grade IV—is a diffusely infiltrating tumor that preliminary affects adults. It is the most common and aggressive form of glioma and is associated with a poor prognosis [2,3]. The incidence of GBM increases with age, with the highest rates observed in individuals aged 75 to 84 years in the United States [2,4]. The standard treatment consists of surgical resection followed by radiotherapy and chemotherapy, typically with oral temozolomide. However, despite aggressive therapy, the median survival time remains under two years, and recurrence is nearly inevitable [5].

Beyond its classical role in coordinating with other immune components to eliminate foreign microorganisms, the complement system is increasingly recognized for its involvement in various pathophysiological processes, including the regulation of the tumor microenvironment and promotion of tumor progression [6,7]. This latter function is particularly relevant in GBM. In GBM, in addition to the tumor cells’ robust DNA repair capacity—which is a major contributor to chemotherapy resistance—the self-renewing capability of therapy-resistant glioma-initiating cells represents another key factor driving treatment failure and tumor recurrence [2,8]. In this context, extensive tissue hypoxia and associated factors such as hypoxia-inducible factor 1 (HIF-1) are known to support the maintenance and tumorigenic potential of glioma-initiating cells. Notably, several components of the complement system, including complement component 3 (C3) and receptors—C3a receptor (C3AR) and C5a receptor C5AR)—are significantly upregulated in GBM in response to hypoxia [9,10].

The complement system is activated by three primary pathways: the classical pathway, triggered by antigen–antibody complexes; the alternative pathway, initiated by permissive surfaces; and the lectin pathway, activated by the binding of pattern-recognizing mannose-binding lectins (MBLs) to carbohydrate ligands on the surface of pathogens [7]. C3 is a central component to all three activation pathways. Upon activation, C3 is cleaved by C3 convertase into C3a and C3b. C3a is subsequently secreted and binds to the C3a receptor (C3AR) on the cell membrane. Similarly, C5 is cleaved by C5 convertase into C5a, which binds to the C5a receptor (C5AR) on the target cells [7,10].

As previously mentioned, complements such as C3, C3AR1, and C5AR1 are upregulated in the tumors–particularly under hypoxic conditions–and contribute to GBM aggressiveness through multiple mechanisms. These include, but are not limited to, maintenance of stemness, promotion of cell proliferation, disruption of barriers to invasion, enhancement of a pro-tumorigenic microenvironment, modulation of immune cell function, and resistance to chemotherapy [10,11,12,13]. Due to their strong association with cancer aggressiveness and poor prognosis, complement-related signaling pathways are attracting increasing interest as potential therapeutic targets [6,10].

Epigenetic modifications, including histone modifications, are recognized as key mechanisms in the development of GBM [14]. Histones, particularly H3 and H4, undergo acetylation and methylation, which can either repress or activate gene transcription. Notably, histone modifications play critical roles in the rapid acquisition of drug resistance in GBM, potentially by contributing to the formation of epigenetically poised cells that undergo chromatin remodeling [4,14]. Although epigenetic alterations that affect histone modifications are known to influence tumor development, prognosis, and cellular characteristics—including the stemness of glioma-initiating cells—their roles in the context of complement activation remain largely unexplored.

Bromodomain and extraterminal domain (BET) proteins are epigenetic readers that recognize acetylated lysine residues on histone tails and regulate gene transcription by recruiting coactivator or corepressor complexes [15,16]. In cancer, particularly GBM, BET proteins—especially BRD4—are often overexpressed and enriched at oncogenic promoters and enhancers, facilitating tumor progression [15,16]. Several BET inhibitors are currently undergoing clinical trials for conditions including cognitive dysfunction, cardiovascular disease, and renal disorders [17,18]. While their specific role in regarding complement activity in GBM remains uncharacterized, BET inhibition has been proposed to mitigate complement-mediated risks associated with cardiovascular diseases and metabolic diseases [19].

Among the histone deacetylases (HDACs), HDAC6—a class IIb member—is notably overexpressed in GBM, including in temozolomide-resistant GBM cells and GBM stem-like tumorspheres [20,21,22]. HDAC6 is predominantly localized in the cytoplasm, where it regulates key mediators of GBM pathogenesis, such as α-tubulin, HSP90, cortactin, and SP1. Through these targets, HDAC6 orchestrates critical cellular processes including cytoskeleton dynamics, cell migration, autophagy, and immune responses [20,23,24]. Notably, HDAC6 inhibition activates DNA damage repair pathways by upregulating cellular stress genes such as DDIT4 (RTP801/Dig2/REDD1) and DDIT3 (CHOP/GADD153), highlighting its potential as a therapeutic target in cancer treatment [25].

Interestingly, a recent study identifies HDAC6 as a valine sensor, with intracellular valine levels tightly regulating its nuclear–cytoplasmic shuttling. Valine deprivation results in the nuclear retention of HDAC6, where it promotes active DNA demethylation, subsequently leading to increased DNA damage. Thus, in response to dietary valine restriction, HDAC6 induces both DNA demethylation and DNA damage, highlighting its potential as a target of cancer therapy [26]. It can be further hypothesized that nutritional factors beyond valine may also influence HDAC6-mediated DNA damage responses.

In this context, nicotinamide adenine dinucleotide (NAD) is an essential cofactor involved in numerous cellular processes, including energy production, cell cycle regulation, and DNA repair, among others [27]. NAD can be synthesized from tryptophan, nicotinamide riboside, or nicotinic acid. Synthesis from tryptophan occurs via the kynurenine pathway (Supplementary Figure S1). Enzymes involved in NAD biosynthesis are often dysregulated in GBM and play critical roles in tumor progression. Interestingly, although the precise mechanisms are not yet fully understood, dysregulation of the kynurenine pathway has also been implicated in immune dysregulation, particularly affecting immune cell activity [28]. However, the extent to which this immune dysregulation contributes to GBM pathophysiology, particularly in relation to GBM cells themselves, remains unclear.

Our preliminary findings indicate that among the key enzymes of the kynurenine pathway, kynureninase (KYNU) is significantly upregulated in GBM patients, and this upregulation closely correlates with the expression of complement system-related genes. Additionally, HDAC6 and BET inhibitors are known to differentially modulate gene expression and have shown promising therapeutic effects in GBM. Based on these observations, we hypothesize that KYNU contributes to the activation of the complement system in GBM. We further propose that the epigenetic regulators, such as HDAC6, may interact with KYNU, forming a potential therapeutic axis to counteract complement-mediated tumorigenicity.

Using publicly available cancer databases and GBM cell lines, we demonstrate that KYNU functions as an upstream regulator of several complement components deregulated in GBM. Notably, HDAC6 silencing upregulates KYNU, which may impair both immunomodulatory and cytotoxic responses. In contrast, KYNU inhibition—particularly when combined with BET-mediated restoration of HDAC6—enhances GBM cell cytotoxicity. These results highlight a previously unrecognized regulatory interaction between metabolic and epigenetic pathways that influence complement signaling, offering new insight into how modulating KYNU and HDAC6 may reshape the tumor immune environment and improve therapeutic responses.

## 2. Results

### 2.1. KYNU Overexpression Is Strongly Correlated with Genes Related to the Complement Cascade in GBM Patients

**Figure 1:**
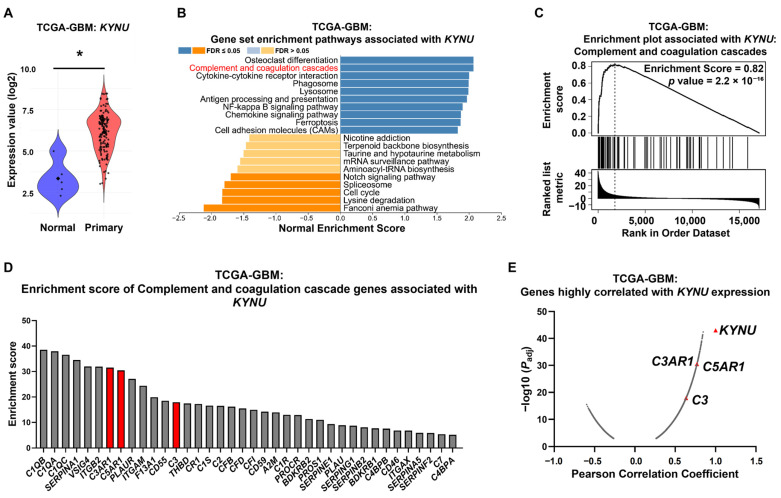
Kynureninase (KYNU) is highly expressed in glioblastoma (GBM) and strongly correlates with complement cascade-related genes. (**A**) KYNU mRNA expression was compared between normal brain tissue (Normal, N = 5) and primary GBM tissue (Primary, N = 154) using TCGA-GBM data accessed via the GDC portal. Differences in mean expression were estimated using nonparametric bootstrap resampling (10,000 iterations). Each dot represents an individual sample; large central points indicate group means. Bootstrapped 95% confidence intervals (CIs) for the mean difference are reported in the main text. Statistical significance was inferred if the 95% CI did not include zero. * *p* < 0.05. (**B**) Gene set enrichment signature pathways associated with KYNU expression in TCGA-GBM samples, categorized by KEGG pathway annotations. (**C**) Gene set enrichment analysis (GESA) and (**D**) enrichment score plot for the complement and coagulation cascade gene set associated with KYNU expression. (**E**) Correlation plot showing genes significantly associated with KYNU expression (*p* < 0.01). Additional details are provided in the Materials and Methods Section.

We first evaluated *KYNU* expression across 24 cancer types available in The Cancer Genome Atlas Program (TCGA) database using the UALCAN web portal [29]. As shown in Supplementary Figure S2A, *KYNU* was upregulated in ten cancer types, and downregulated in four. For GBM, bootstrap analysis revealed that the expression of *KYNU* was significantly elevated in GBM samples compared to normal brain tissue (mean difference = 2.801, 95% CI = 1.747 to 3.639) (Figure 1A). This upregulation of KYNU indicates its potential as a targetable molecule in GBM, and possibly other cancers. Further analysis of the TCGA-GBM dataset revealed that although *KYNU* overexpression was not significantly associated with overall survival, it was linked to a shortened progression-free interval (Supplementary Figure S2B,C).

To identify genes and pathways associated with high *KYNU* expression in GBM patients, we performed gene set enrichment analysis (GSEA) using gene categories defined in the Kyoto Encyclopedia of Genes and Genomes (KEGG) pathway annotations. Among the enriched pathways, the complement and coagulation cascades ranked second, with normalized enrichment score of 2.07, indicating significant upregulation in KYNU-high tumors (Figure 1B–D). Moreover, the expressions levels of several complement-related genes showed strong positive correlations with *KYNU* expression (Figure 1E).

Although the precise mechanisms underlying KYNU overexpression and its role in GBM progression remain unclear, analysis of larger datasets—including information on genetic alterations in KYNU and related pathways—will be necessary to better understand its upstream regulation and prognostic significance. Nonetheless, its strong correlation with complement signaling suggests that KYNU overexpression may contribute to dysregulation of this pathway in GBM.

### 2.2. C3, C3AR1, and C5AR1 Along with KYNU Are Overexpressed in Selected GBM Cell Lines

**Figure 2:**
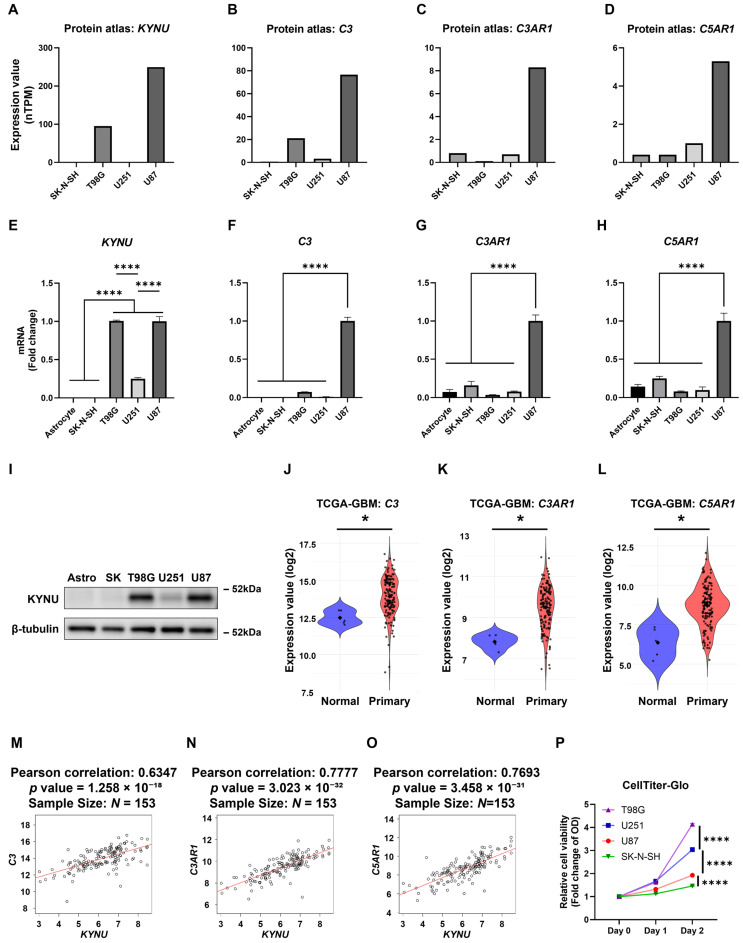
KYNU, C3, C3AR1, and C5AR1 are expressed in GBM cell lines. (**A**–**D**) Gene expression levels (normalized transcripts per million, nTPM) for *KYNU*, *C3*, *C3AR1*, and *C5AR1* across various GBM cell lines, obtained from the Human Protein Atlas and GTEx. (**E**–**H**) Basal mRNA expression in selected cell lines was measured by qRT-PCR (N = 3), and presented as fold change relative to U87. (**I**) Protein levels were assessed by immunoblotting, with β-tubulin used as a loading control. (**J**–**L**) Comparison of gene expression between normal brain tissue (Normal, N = 5) and primary GBM tissue (Primary, N = 154) using TCGA-GBM data accessed via the GDC portal. Differences in mean expression were estimated using nonparametric bootstrap resampling (10,000 iterations). Each dot represents an individual sample; large central points indicate group means. Bootstrapped 95% confidence intervals (CIs) are reported in the main text. Statistical significance was inferred if the 95% CI did not include zero. * *p* < 0.05. (**M**–**O**) Pearson correlation analysis between *KYNU* and *C3*, *C3AR1*, or *C5AR1* in TCGA-GBM samples. (**P**) Cell viability was assessed using the CellTiter-Glo assay over 48 h (N = 4). Statistical analysis: One-way ANOVA with Tukey’s post hoc test was used for panels (**E**–**H**); two-way ANOVA with Tukey’s post hoc test was applied for panel (**P**). **** *p* < 0.0001. Bars represent mean + SEM.

To further investigate KYNU expression in GBM, we queried the Human Protein Atlas database to assess its levels across various GBM cell lines. KYNU was highly expressed in U87 and T98G cells, while its expression was nearly undetectable in SK-N-SH and U251 cells (Figure 2A). Given the known variability in complement protein expression across cell-types, we next examined the expression of complement components in GBM cell lines. Among 38 complement- and coagulation cascade-related genes significantly associated with *KYNU* expression (Figure 1D), *C3*, *C3AR1*, and *C5AR1* were consistently expressed across most cell lines—except for *C3*, which was absent in SK-N-SH cells. Notably, all three genes were abundantly expressed in U87 cells (Figure 2B–D).

To validate these findings, we cultured selected GBM cell lines and quantified mRNA expression relative to astrocytes. As shown in Figure 2E–H, both *KYNU* and *C3* were absent in astrocytes and SK-N-SH cells, but were highly expressed in U87 cells. Western blot analysis further confirmed high KYNU protein levels in T98G and U87 cells, moderate levels in U251, and no detectable expression in astrocytes or SK-N-SH cells (Figure 2I). We speculate that the gene expression profile of SK-N-SH cells, which closely resembles that of astrocytes, may be attributed to their origin from a bone marrow aspirate of a child with metastatic neuroblastoma, rather than from primary GBM.

Consistent with elevated *KYNU* expression in GBM (Figure 1A), the complement components *C3* (mean difference = 1.440, 95% CI = 0.937 to 1.886), *C3AR1* (mean difference = 1.664, 95% CI = 1.333 to 2.025), and *C5AR1* (mean difference = 2.337, 95% CI = 1.441 to 3.255), were significantly upregulated in GBM tissues (Figure 2J–L). These genes also showed strong positive correlations with *KYNU* expression in GBM patient samples (Figure 2M–O). Interestingly, despite high KYNU expression, U87 cell proliferation was markedly slower—comparable to that of KYNU-deficient SK-N-SH cells (Figure 2P). Whether KYNU directly influences cell proliferation remains to be determined and warrants further investigation. Nonetheless, these findings support the notion that KYNU may regulate the expression of C3, C3AR1, and C5AR1 in GBM cells, thereby contributing to complement-mediated tumorigenic processes.

### 2.3. KYNU Is an Upstream Regulator of C3, C3AR1, and C5AR1

**Figure 3:**
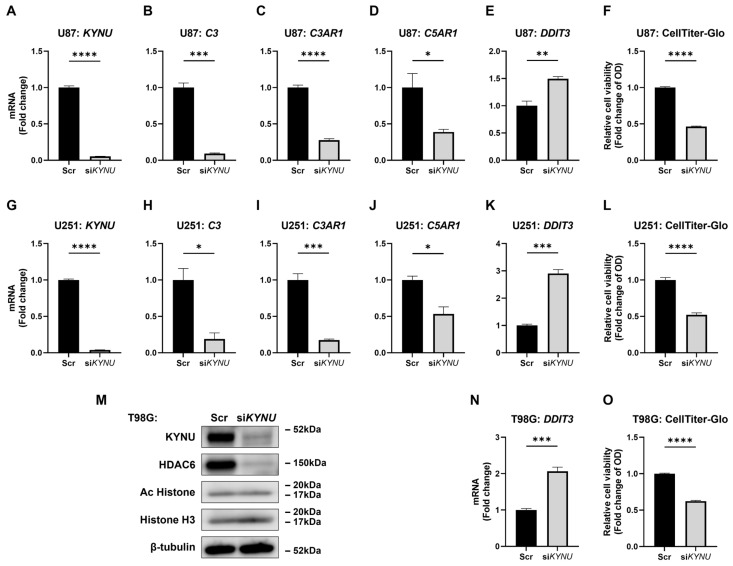
KYNU acts as an upstream regulator of complement genes. U87, U251, and T98G glioblastoma cells were transfected with scrambled control siRNA (Scr) or *KYNU*-specific siRNA (si*KYNU*). Four days post-transfection: (**A**–**K**,**N**) mRNA expression of target genes was quantified by qRT-PCR (N = 3); (**M**) protein levels were assessed by immunoblotting, with β-tubulin used as a loading control; (**F**,**L**,**O**) cell viability was measured using the CellTiter-Glo assay (N = 4). Statistical analysis: Two-tailed unpaired Student’s *t*-test was applied to all panels except (**M**). * *p* < 0.05; ** *p* < 0.01; *** *p* < 0.001; **** *p* < 0.0001. Bars represent mean + SEM.

To investigate whether KYNU is an upstream regulator of complement gene expression, we silenced *KYNU* in U87, U251, and T98G cells. Astrocytes and SK-N-SH cells were excluded from further analysis due to their negligible KYNU expression. KYNU silencing effectively reduced both mRNA and protein levels of KYNU (Figure 3A,G,M), and led to marked downregulation of *C3*, *C3AR1*, and *C5AR1* (Figure 3B–D,H–J). In addition to complement components, *KYNU* silencing also decreased the expression of key immunomodulatory genes, including *interleukin 6 (IL6)* [30], *IL10RB* [31], and *MAP3K8*, a prognostic biomarker of immunotherapy [32]. Expression of *vascular endothelial growth factor (VEGF) A* [2], a hypoxia-response gene, and *CD55* (also known as decay-accelerating factor), a complement regulatory protein was also significantly reduced [9] (Supplementary Figure S3A–E). Functionally, *KYNU* silencing induced the cellular stress marker *DDIT3*, decreased total cell numbers across all three GBM cell lines (Figure 3E,F,K,L,N,O), and reduced colony formation in U87 cells (Supplementary Figure S3F).

To explore the effect of pharmacological targeting of KYNU on this pathway, we treated GBM cells with apabetalone (RVX-208), a BET inhibitor [17,18]. Apabetalone suppressed *KYNU*, *C3* and *C3AR1* expression; while paradoxically upregulating *C5AR1* (Supplementary Figure S4A–D,G–J). Despite this inconsistency, apabetalone increased *DDIT3* expression and reduced cell viability in a dose-dependently manner (Supplementary Figure S4E,F,K,L). Together, these findings confirm that *KYNU* is a functionally targetable molecule whose inhibition suppress complement activation, induces cellular stress response, and impairs GBM cell viability. However, the upregulation of C5AR1 following apabetalone treatment suggests that additional regulatory mechanisms, independent of KYNU may influence the expression of certain complement genes, particularly C5AR1.

### 2.4. HDAC6 Inhibition Upregulates Some Complement Components

**Figure 4:**
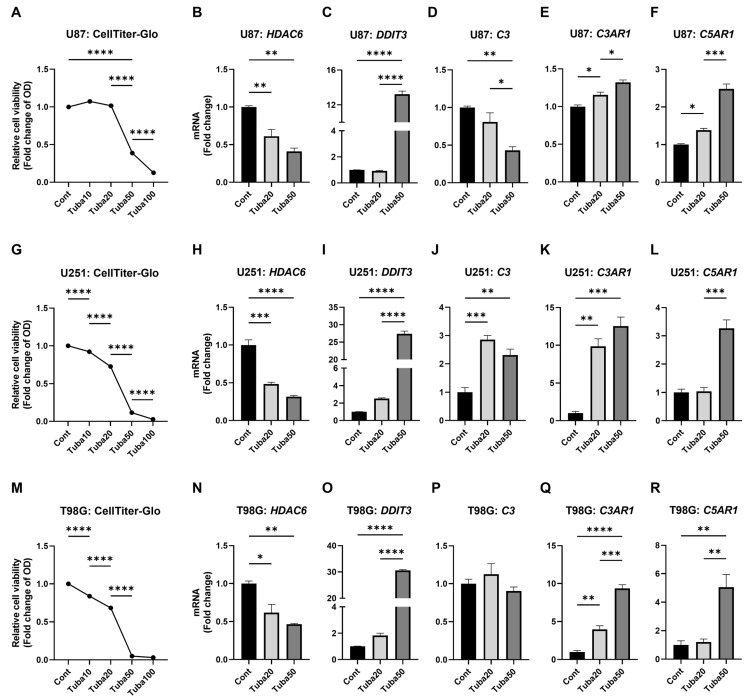
HDAC6 inhibition regulates some complement genes. U87, U251, and T98G glioblastoma cells were treated with DMSO (Cont) or tubastatin (10, 20, 50, or 100 μM). (**A**,**G**,**M**) Cell viability was assessed using the CellTiter-Glo assay after 48 h (N = 4). (**B**–**F**,**H**–**L**,**N**–**R**) mRNA expression of target genes was measured by qRT-PCR after 24 h (N = 3). Statistical analysis: Two-way ANOVA with Tukey’s post hoc test was used for panels (**A**,**G**,**M**); one-way ANOVA with Tukey’s post hoc test was applied to panels (**B**–**F**,**H**–**L**,**N**–**R**). * *p* < 0.05; ** *p* < 0.01; *** *p* < 0.001; **** *p* < 0.0001. Bars represent mean + SEM.

Analysis of TCGA data revealed a modest but statistically non-significant increase in HDAC6 expression in GBM tissues (mean difference = 0.172, 95% CI = −0.205 to 0.414; Supplementary Figure S5A). Consistent with this, HDAC6 was found to be ubiquitously expressed across GBM cell lines (Supplementary Figure S5B,C). In parallel, global histone H3 acetylation was elevated in T98G, U251, and U87 cells (Supplementary Figure S5D). Given HDAC6′s widespread expression in both malignant and non-malignant brain tissues—and the growing interest in HDAC6 as a therapeutic target in cancer [22,33]—we investigated the effects of its selective inhibition using tubastatin [34]. As anticipated from previous studies, tubastatin treatment led to dose-dependent downregulation of *HDAC6*, robust upregulation of the stress gene *DDIT3*, and significantly reduced cell viability in all tested GBM cells (Figure 4A–C,G–I,M–O).

Interestingly, the impact of tubastatin on complement genes was variable. *C3* expression showed inconsistent regulation across cell lines (Figure 4D,J,P). However, tubastatin consistently upregulated *C3AR1* and *C5AR1* across all three U87, U251, and T98G cells (Figure 4E,F,K,L,Q,R). These results suggest that, while HDAC6 inhibition exerts antitumor effects via stress induction and viability reduction, the concurrent upregulation of at least *C3AR1* and *C5AR1* may inadvertently activate complement signaling, potentially counteracting its therapeutic benefits in complement-rich GBM microenvironments. Supporting this notion, tubastatin’s cytotoxic efficacy was notably less pronounced in U87 cells, which express high levels of complement genes (Supplementary Figure S6).

### 2.5. KYNU Inhibition Attenuates HDAC6-Mediated Dysregulation of Complement Genes

While both KYNU and HDAC6 activate stress response and suppress GBM cell viability, they exert opposing effects on the expression of complement genes. Specifically, *KYNU* silencing led to the downregulation of complement components, whereas *HDAC6* inhibition using tubastatin resulted in their upregulation. To investigate the potential crosstalk between these two regulatory pathways, we evaluated the combined effects of KYNU and HDAC6 inhibition on complement gene expression and cell viability.

**Figure 5:**
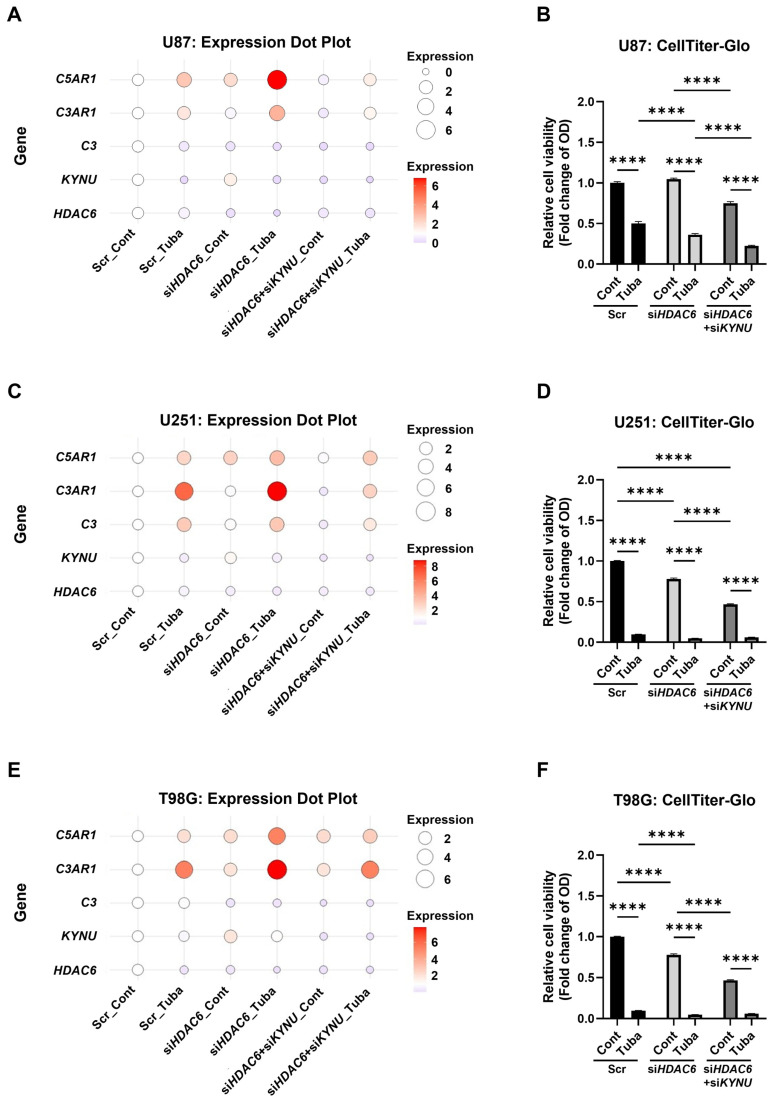
KYNU inhibition attenuates HDAC6-mediated dysregulation of complement components. U87, U251, and T98G glioblastoma cells were transfected with scrambled control siRNA (Scr), *HDAC6* specific siRNA (si*HDAC6*), or combined *HDAC6* and *KYNU* siRNAs (si*HDAC6* + si*KYNU*). Three days post-transfection, cells were treated with DMSO (Cont) or tubastatin (50 μM; Tuba). (**A**,**C**,**E**) mRNA expression of target genes was assessed by qRT-PCR 24 h after tubastatin treatment. Data are visualized as dot plots, where the color intensity and size of each dot represent the mean relative expression level. Expression values were normalized to the Scr_Cont group (set as 1.0) and represent the mean of three biological replicates (N = 3). The blue-to-red gradient indicates downregulation to upregulation relative to baseline. Additional data are provided in Supplementary Figure S7. (**B**,**D**,**F**) Cell viability was measured using the CellTiter-Glo assay 48 h after tubastatin treatment (N = 4). Statistical analysis: Two-way ANOVA with Tukey’s post hoc test was applied to panels (**B**,**D**,**F**). **** *p* < 0.0001. Bars represent mean + SEM.

Both HDAC6 knockdown and tubastatin treatment significantly reduced HDAC6 mRNA and protein levels. In addition, tubastatin increased global histone H3 acetylation, consistent with its role as an epigenetic modulator (Figure 5A,C,E; Supplementary Figures S7A,F,K and S8). Notably, tubastatin also enhanced histone acetylation even in HDAC6 knockout cells, suggesting potential off-target effects, possibly through inhibition of other histone deacetylases such as Class I HDACs. Further investigation is warranted to elucidate the effects of tubastatin on HDACs beyond HDAC6 [35,36].

As observed previously, *C3* expression varied across cell lines, being downregulated in U87 and T98G cells but upregulated in U251 cells (Figure 5A,C,E; Supplementary Figure S7C,H,M), indicating the cell-specific regulatory mechanisms. Moreover, HDAC6 inhibition consistently upregulated *C5AR1* and, to a lesser extent, *C3AR1*, with tubastatin treatment further enhancing their expression (Figure 5A,C,E; Supplementary Figure S7D,E,I,J,N,O), suggesting that complement receptor regulation may be influenced by multiple layers of control.

Interestingly, *HDAC6* knockdown increased *KYNU* mRNA expression, while tubastatin treatment led to its downregulation (Figure 5A,C,E; Supplementary Figure S7B,G,L). This differential regulation suggests that HDAC6 may modulate KYNU expression. The upregulation of KYNU by HDAC6 knockdown could, in turn, contribute to the induction of C3AR1 and C5AR1, potentially counteracting the cytotoxic effects of HDAC6 inhibition. Supporting this notion, the combined treatment of tubastatin with simultaneous knockdown of *KYNU* and *HDAC6* markedly attenuated the expression of *C3*, *C3AR1*, and *C5AR1*, and further reduced GBM cell viability (Figure 5; Supplementary Figure S7). These findings highlight the therapeutic potential of co-targeting KYNU and HDAC6 to suppress complement-mediated resistance in GBM. However, given the known heterogenicity of GBM and the variability in basal complement expression across tumors, the therapeutic efficiency of this strategy may vary among patients.

### 2.6. Inhibition of KYNU-HDAC6-Mediated Complement Activation Reduces GBM Cell Viability

**Figure 6:**
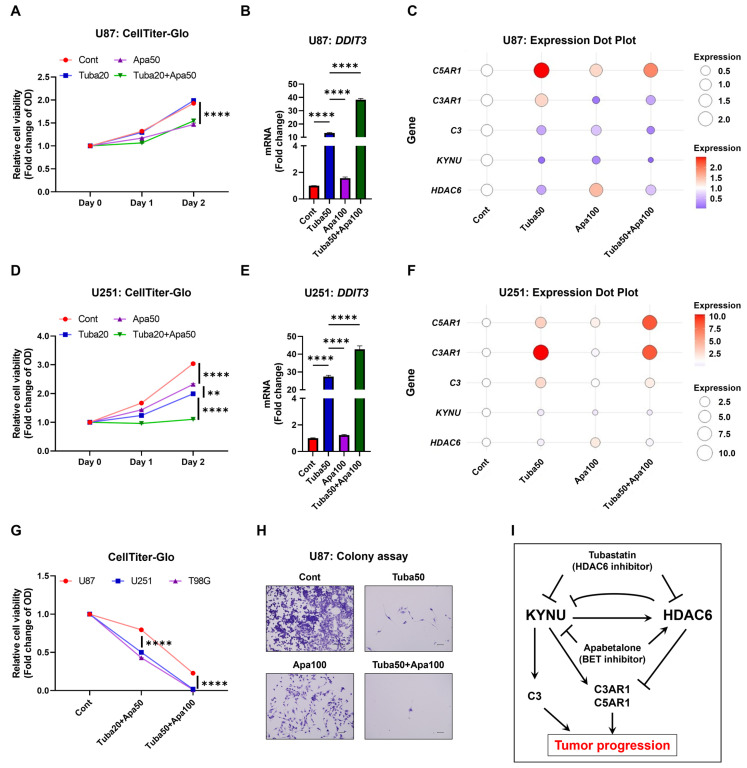
Inhibition of KYNU-HDAC6-mediated complement activation reduces GBM cell viability. U87 and U251 glioblastoma cells were treated with DMSO (Cont), tubastatin (20 μM), apabetalone (50 μM), or their combinations for the indicated durations. (**A**,**D**) Cell viability was assessed using the CellTiter-Glo assay at the specified time points (N = 4). (**B**,**E**) mRNA expression of target genes was measured by qRT-PCR after 24 h of treatment (N = 3). (**C**,**F**) Dot plot visualization of gene expression following 24 h treatment. Dot size and color intensity reflect the mean relative expression level of each gene. Expression values were normalized to the Cont group (set as 1.0) and represent the average of three biological replicates (N = 3). The blue-to-red gradient indicates fold changes below and above baseline. Additional details are provided in Supplementary Figure S9. (**G**) U87, U251, and T98G cells were treated with DMSO (Cont), 20 μM tubastatin + 50 μM apabetalone (Tuba20 + Apa50), or 50 μM tubastatin + 100 μM apabetalone (Tuba50 + Apa100) for 48 h, and cell viability was assessed using the CellTiter-Glo assay (N = 4). (**H**) Representative crystal violet-stained colony formation images after 7 days of treatment. (**I**) Schematic summary of the proposed mechanism. Statistical analysis: Two-way ANOVA with Tukey’s post hoc test was used for panels (**A**,**D**,**G**); one-way ANOVA with Tukey’s post hoc test was used for panels (**B**,**E**). ** *p* < 0.01; **** *p* < 0.0001. Bars represent mean + SEM. (**H**) Scalebar = 100 μm.

Given that HDAC6 inhibition paradoxically upregulates KYNU, potentially diminishing the cytotoxic efficacy of tubastatin, and that the BET inhibitor apabetalone suppresses KYNU expression, we evaluated the combined effects of tubastatin and apabetalone across a range of concentrations. Based on preliminary screening, we selected doses that demonstrated optimal efficacy. Tubastatin alone effectively reduced viability in U251 cells, which express low levels of complement components, but had limited efficacy in U87 cells, characterized by high complement expression. In contrast, apabetalone alone significantly suppressed cell viability in both cell lines, and its combination with tubastatin led to enhanced cytotoxicity (Figure 6A,D).

Consistently, apabetalone augmented tubastatin-induced expression of the stress-response gene DDIT3, indicating an amplified cellular stress response (Figure 6B,E). Furthermore, apabetalone downregulated KYNU, C3, and C3AR1, while simultaneously upregulating HDAC6 (Figure 6C,F; Supplementary Figure S9B–E,H–K). Importantly, at higher concentrations, both agents displayed robust cytotoxic effects across all tested GBM cell lines, independent of their baseline KYNU or complement expression levels (Figure 2 and Figure 6G). These findings suggest that apabetalone can counteract HDAC6 inhibition-induced KYNU upregulation and enhance the therapeutic efficacy of tubastatin. Thus, concurrent targeting of KYNU and HDAC6 may represent a promising combinatorial strategy for overcoming complement-mediated resistance in GBM.

## 3. Discussion

Resistance to treatment remains a major challenge in GBM. Multiple mechanisms underlie this resistance, including robust DNA repair capabilities driven by genetic and epigenetic alterations, the self-renewing and tumorigenic potential of glioma-initiating cells, the emergence of drug resistance or highly invasive subpopulations of tumor cells, and the presence of an immunosuppressive tumor microenvironment [2,8]. Within this tumor microenvironment, activation of the complement system has been shown to promote tumor growth through direct autocrine signaling, independent of its classical immunomodulatory roles [7,10]. Notably, several studies have emphasized a strong association between hypoxia and the activation of local complement signaling in GBM [9,10,12]. In this study, we identify a novel regulatory role of KYNU, an intermediate enzyme in the kynurenine pathway of NAD synthesis, in the expression of complement components in GBM. This finding expands our understanding of how metabolic pathways intersect with immune signaling in the tumor microenvironment and highlights KYNU as a potential therapeutic target for modulating complement activation in GBM.

In the context to GBM, upregulation of several complements—such as C1q, C3, and C5aR1—has been reported in patient serum, tumor tissues and GBM cell lines [10,11,12]. Given the activation of the complement system in GBM, immunotherapy has been proposed as a potential therapeutic strategy. However, unlikely in many other cancers, immunotherapy using C1 inhibitor in a murine intracranial tumor model failed to produce any significant survival benefit [37]. This likely reflects the inherently immunosuppressive microenvironment characteristic of GBM, which contributes to its resistance to immune-based therapies [2]. Therefore, there is an urgent need to identify novel therapeutic targets and small molecules capable of crossing the blood–brain barrier.

In this study, we demonstrate that C3, C3AR1, and C5AR1 are robustly expressed in GBM cells, indicating potential tumor cell-specific functions of these complement components. Notably, we identify kynureninase (KYNU) as an upstream regulator of their expression. KYNU inhibition led to the downregulation of C3, C3AR1, and C5AR1, along with decreased expression of key hypoxia-associated (CD55, VEGFA) and inflammatory (IL6, IL10RB, MAP3K8) markers. In parallel, KYNU silencing significantly upregulated the cellular stress response gene DDIT3 and enhanced cytotoxicity, indicating activation of pro-apoptotic signaling (Figure 3; Supplementary Figure S3). These findings suggest that targeting KYNU may not only suppress complement-driven signaling but also enhance stress-induced cytotoxicity, thereby attenuating glioblastoma progression.

C3 is a central component of the complement system and plays a pivotal role in the classical activation pathway. Elevated expression of both C3 and its receptor C3AR1 has been associated with poor survival outcomes in patients with glioma, particularly in IDH-wild-type GBM [9]. The C3a/C3AR1 axis is critically involved in promoting glioma cell self-renewal, as well as in the polarization of macrophages and microglia, and it has been implicated in facilitating leptomeningeal metastasis [9,11]. Notably, antagonism of C3AR with the small-molecule inhibitor SB290157 was shown to reduce sphere formation in U3082MG glioma cells, indicating that C3AR signaling contributes to glioma stem-like properties. Furthermore, SB290157 extended survival in glioma-bearing mice, both as a monotherapy and in combination with radiotherapy, and also prevented leptomeningeal metastasis in non-GBM mouse models [9,11].

Similarly, C5AR1 is markedly upregulated in GBM compared to normal brain tissue, and its activation has been shown to promote cell invasion, migration, and tumor growth, ultimately leading to reduced survival [38]. While not yet extensively studied in GBM, C5AR1 has been validated as a druggable target in other cancer models. For instance, pharmacological inhibition using the selective antagonist PMX205 enhanced radiotherapy efficiency in colorectal tumors, including those with immunosuppressive features [39]. These findings underscore the functional relevance of C3, C3AR1, and C5AR1 signaling within the tumor microenvironment and highlight their potential as therapeutic targets.

Consistent with these observations, our study demonstrates that KYNU silencing or its pharmacological inhibition via the BET inhibitor apabetalone significantly downregulated complement gene expression, elevated the stress response marker DDIT3, and reduced GBM cell viability (Figure 5 and Figure 6). Although in vivo validation remains to be conducted, we speculate that KYNU inhibition may also influence modulation in the tumor microenvironment—particularly complement activation—and could potentially suppress metastatic progression.

Among various epigenetic alterations, histone modifications are recognized as a key mechanism driving the development of GBM [14]. These modifications are critically involved not only in malignant transformation and tumor progression but also in contributing to the functional heterogeneity and hierarchical maintenance of glioma-initiating cells [23]. Furthermore, aberrant histone modification patterns have emerged as important prognostic markers in GBM patients [2]. Consequently, epigenetic regulation—particularly through histone modifications—has attracted considerable attention in GBM research, offering promising avenues for both biomarker discovery and therapeutic development.

In this context, HDAC6 is frequently overexpressed in GBM and contributes significantly to tumor progression. Although primarily a cytoplasmic enzyme, HDAC6 deacetylates several non-histone substrates and can translocate into the nucleus to influence gene expression through interactions with transcription factors [40]. Notably, HDAC6 has been shown to promote DNA damage repair by regulating the Sp1 transcription factor, thereby facilitating drug resistance and enhancing GBM cell survival [21]. Pharmacological inhibition of HDAC6 using agents such as MPT0B291 has been shown to downregulate the expression of DNA damage response genes, induce DNA damage, and impair homologous recombination repair in GBM cells, including those resistant to temozolomide. Additionally, several other selective HDAC6 inhibitors—such as tubastatin, ACY-1215, and CAY10603—have been reported to overcome temozolomide resistance through various mechanisms, including suppression of epidermal growth factor receptor (EGFR) expression, reduction in clonogenicity and migration, attenuation of temozolomide-induced endoplasmic reticulum stress tolerance, and enhancement of temozolomide-induced apoptosis [20,23,33,34].

The HDAC6 inhibitor tubacin has been shown to promote cell death through the intrinsic apoptotic pathway by inducing the expression of cellular stress genes such as DDIT4 (RTP801/Dig2/REDD1) and DDIT3 (CHOP/GADD153), thereby triggering DNA damage in various cancer cells [25]. Tubacin has also been reported to enhance the cytotoxic effects of the pan-HDAC inhibitor SAHA (vorinostat) [25]. In our study, tubastatin, another selective HDAC6 inhibitor, similarly upregulated DDIT3 expression, and reduced GBM cell viability. However, it also upregulated complement receptors *C3AR1* and *C5AR1* (Figure 4; Supplementary Figure S6). Notably, the inconsistent regulation of C3 among the different GBM cell lines used in this study (U87, U251, and T98G) suggests possible off-target effects or the involvement of counteracting regulatory mechanisms specific to C3 expression. These findings underscore the need for further investigation using additional GBM cell lines with varying baseline levels of complement components. Such studies will be essential to fully elucidate the potential off-target actions of tubastatin A and to identify additional regulatory factors beyond HDAC6 that may be influenced by its treatment [35,36].

We assume that in the complement-rich, immunosuppressive microenvironment of GBM, this paradoxical upregulation of complement receptors may dampen the therapeutic efficacy of HDAC6 inhibitors. This underscores the need for strategies that co-target epigenetic and immune-regulatory pathways. Indeed, we found that KYNU silencing not only downregulated complement gene expression but also amplified the cytotoxic effect of tubastatin (Figure 5), suggesting a synergistic interaction between KYNU and HDAC6. These findings indicate that targeting the KYNU-HDAC6 axis may offer enhanced therapeutic potential in GBM by addressing both tumor-intrinsic and microenvironmental resistance mechanisms.

Of particular interest, a recent study identifies human HDAC6 as a metabolic sensor of the essential-branched chain amino acid valine. Under valine-deprived conditions, HDAC6 is retained in the nucleus, where it activates DNA demethylation by binding to and deacetylating TET2, subsequently promoting DNA damage. This discovery underscores the therapeutic potential of dietary valine restriction in cancer treatment. Our findings extended this metabolic link by demonstrating that KYNU, a key enzyme in the kynurenine pathway of NAD^+^ biosynthesis, can also regulate the expression of HDAC6. Given HDAC6′s diverse roles in DNA repair, epigenetic regulation, and GBM progression, further studies are needed to elucidate the transcriptional and post-transcriptional mechanisms through which KYNU modulates HDAC6 and complement gene expression. A deeper understanding of this axis may open new avenues for integrated metabolic and epigenetic therapies targeting complement-related resistance mechanisms in GBM.

The kynurenine pathway of NAD^+^ biosynthesis is frequently dysregulated in GBM. Key enzymes such as indoleamine 2,3-dioxygenase (IDO1) and tryptophan 2,3-dioxygenase (TDO2) are commonly overexpressed in human glioma cells, resulting in elevated production of kynurenine. This metabolite is further converted to quinolinic acid, a known neurotoxin. Kynurenine also acts as an endogenous ligand for the aryl hydrocarbon receptor (AhR), promoting tumor immune evasion and progression [41]. Therapeutic strategies targeting IDO1/TDO2–kynurenine–AhR axis aim to reduce immunosuppression and neurotoxicity by inhibiting kynurenine synthesis. However, these approaches have shown limited efficacy in preclinical and clinical settings [41], with minimal cytotoxic effects in vitro [42], likely due to their reliance on immune activation rather than direct cell killing [41].

Although we focused on complement activation in this study, KYNU, a rate-limiting enzyme downstream of kynurenine, emerges as a distinct and potentially more effective therapeutic target. As a key regulator of quinolinic acid and NAD^+^ production, KYNU inhibition may influence not only complement signaling but also metabolic stress and immune modulation. Further investigation into KYNU-specific regulatory mechanisms and the roles of kynurenine pathway metabolites, including NAD^+^, could uncover novel therapeutic opportunities for GBM beyond upstream kynurenine enzymes.

Finally, to explore the therapeutic potential of targeting the KYNU–HDAC6–complement axis, we investigated the effects of the BET inhibitor apabetalone, which has shown promise in GBM by modulating tumor-intrinsic pathways, enhancing temozolomide sensitivity particularly in MGMT-unmethylated cases, and overcoming resistance mechanisms when used in combination therapies [15,16]. In our study, apabetalone suppressed KYNU expression while restoring HDAC6 levels, and its combination with the HDAC6 inhibitor tubastatin resulted in a synergistic increase in the cellular stress marker DDIT3 and a more pronounced cytotoxic effect across GBM cell lines. These findings support the functional significance of the KYNU–HDAC6–complement axis and suggest that combined BET and HDAC6 inhibition may effectively counteract compensatory pathways that undermine the efficacy of monotherapies. This combinatorial approach offers a mechanistically supported strategy to overcome complement-mediated resistance in GBM and supports further investigation of epigenetic modulation integrated with immune regulation in GBM therapy.

## 4. Materials and Methods

### 4.1. Public Dataset Acquisition and Data Processing

We retrieved and analyzed KYNU mRNA expression across normal tissues and 24 cancer types using data from The Cancer Genome Atlas (TCGA) via the UALCAN portal (https://ualcan.path.uab.edu/index.html, accessed on 10 January 2025) [29]. For glioblastoma multiforme (TCGA-GBM) gene expression analysis, we accessed data from the Genomic Data Commons (GDC) through UCSC Xena (https://xena.ucsc.edu/, accessed on 10 January 2025), and performed subsequent analysis in RStudio (version 2025.05.1 Build 513). Expression levels of *KYNU*, *HDAC6*, *C3*, *C3AR1*, and *C5AR1* were retrieved form 154 GBM samples and 5 normal brain samples.

To compare gene expression between glioblastoma (GBM) and normal brain tissues, we employed nonparametric bootstrap resampling approach (10,000 iterations) to estimate the mean difference and 95% confidence intervals for each gene. Given the unequal sample sizes and potential non-normality of expression data, we used an ordinary nonparametric bootstrap method implemented in R (boot package, version 1.3-31). Group labels (“Normal” and “Primary”) were randomly resampled with replacement, and the difference in group means (Primary − Normal) was calculated to generate a distribution of bootstrap distribution of differences. The 95% percentile confidence intervals were derived accordingly. Patient survival data were also obtained through UCSC Xena.

To identify genes highly correlated with KYNU in the TCGA-GBM cohort, we used LinkedOmics portal (https://www.linkedomics.org/admin.php, accessed on 8 February 2025) [43]. We utilized gene expression data obtained via the HiSeq RNA platform (Firehose_RSEM_log2) and conducted Pearson correlation analysis between *KYNU* and all other genes within the same dataset. Gene set enrichment analysis (GSEA) was performed with the LinkInterpreter module, based on KEGG pathway annotations with the following default parameters: Rank Criteria (from LinkFinder Result): *p*-Value, Data is Signed ranked; Minimum Number of Genes (Size): 3; and Simulations: 500). Resulting data were downloaded and further analyzed in GraphPad Prism 9.0.0 (GraphPad Software, Boston, MA, USA). Finally, the expression levels of KYNU and complement-/coagulation cascade-related genes across various GBM cell lines were evaluated using consensus transcriptomic datasets from the Human Protein Atlas (HPA) and GTEx projects, using normalized transcripts per million (nTPM) values (https://www.proteinatlas.org, accessed on 8 February 2025) [44,45].

### 4.2. Cell Lines, Culture, and Reagents for Major Interventions

Human glioblastoma multiforme (GBM) cell lines SK-N-SH, T98G, U251, U87, and human astrocytes (kindly provided by Prof. Nishiyama, Kagawa Medical University) were used in this study. Cells were maintained in Dulbecco’s Modified Eagle Medium (DMEM; D5796, Sigma-Aldrich, St. Louis, MO, USA) supplemented with 10% fetal bovine serum (FBS; S1810-500, Biowest, Lakewood Ranch, FL, USA) and Penicillin–Streptomycin–Glutamine solution (06168-34, Nacalai Tesque, Kyoto, Japan). Cultures were incubated at 37 °C under 95% air/5% CO_2_ and passaged at 50–60% confluency. For interventions, cells were treated with apabetalone (RVX-208) and tubastatin (Tubastatin A) (catalog numbers 16424 and 15785, respectively; Cayman Chemical, Ann Arbor, MI, USA). Control groups received equivalent volumes of dimethyl sulfoxide (DMSO; 13408-64, Nacalai Tesque, Kyoto, Japan).

### 4.3. Gene Silencing Using siRNA Transfection

Gene silencing was performed as previously described [46]. Briefly, cells were seeded in 24-well plates at 500 µL complete medium per well and transfected with 50 nM siRNA targeting *KYNU* (HSS113244) or *HDAC6* (HSS173319) (Thermo Fisher Scientific, Waltham, MA, USA), or with a nonspecific negative control siRNA (Medium GC Duplex #2, Thermo Fisher Scientific, 465372). Transfections were conducted using 1.5 µL Lipofectamine RNAiMAX reagent (13778, Thermo Fisher Scientific) diluted in 100 µL Opti-MEM I Reduced Serum Medium (31985062, Thermo Fisher Scientific). Culture medium was replaced 24 h post-transfection, and all downstream experiments were performed 48 h after transfection.

### 4.4. Total RNA Extraction, cDNA Synthesis, and qRT PCR

Following appropriate interventions, total RNA was extracted, and qRT-PCR was performed as previously described [47,48]. RNA was isolated using TRIzol reagent (15596018, Thermo Fisher Scientific, USA) according to the manufacturer’s protocol. First-strand cDNA synthesis was carried out using the PrimeScript RT reagent Kit (RR037A, Takara Bio, Shiga, Japan). Quantitative PCR was performed on a 7500 Fast Real-Time PCR System (Applied Biosystems, Tokyo, Japan) using PowerTrack SYBR Green Master Mix (A46109, Thermo Fisher Scientific, Vilnius, LT, USA). For each gene, relative expression values were normalized to the corresponding control groups, which was set as 1.0. Each condition represents the mean of three biological replicates. For visualization, gene expression values were imported into RStudio (version 2025.05.1 Build 513) and reshaped using the pivot_longer() function from the tidyverse package. Dot plots were generated using ggplot2, where dot size and color both reflect the expression level of each gene. The color scale was defined using scale_fill_gradient2() with a midpoint of 1 (the control baseline), and axis labels were formatted for clarity. Primer sequences used in this study are listed in Supplementary Table S1.

### 4.5. SDS-PAGE and Western Blotting

Whole-cell lysates were prepared using RIPA buffer (89900, Thermo Fisher Scientific, USA) following the manufacturer’s instructions, with minor modifications as previously described [49]. Proteins were separated on 7.5% polyacrylamide gels (TGX FastCast Acrylamide Kit, 1610171, Bio-Rad Laboratories, Hercules, CA, USA) and transferred onto nitrocellulose membranes (Immobilon-P, IPVH00005, Merck Millipore, Cork, Ireland). The following primary antibodies were used: anti-trimethyl-histone H3 (H3K9me3) (Lys9) and anti-histone H3 (D1H2) (13969 and 4499, respectively; Cell Signaling Technology, Danvers, MA, USA); anti-KYNU and anti-HDAC6 (11796-1-AP and 67250-1-Ig, respectively; Proteintech Group, Rosemont, IL, USA); and anti-β-tubulin (2128, Cell Signaling Technology, USA). Chemiluminescent signals were detected using an Odyssey Fc Imaging System (LI-COR Biosciences, Lincoln, NE, USA) and analyzed with Odyssey Application software (Image Studio Lite version 5.2, LI-COR, Tokyo, Japan) as described previously [47,49].

### 4.6. Cell Viability Assay

Cells were seeded in 96-well plates, and treatments were applied either the following day or 48 h after siRNA transfection. Cell viability was assessed using the CellTiter-Glo Luminescent Cell Viability Assay (Promega, Madison, WI, USA) according to the manufacturer’s instructions. Luminescence was measured with a TECAN Spark Plate Reader (Tecan Austria GmbH, Grödig, Austria) as previously described [46].

### 4.7. Colony Formation Assay

Following treatments, colonies were fixed and stained with 0.1% crystal violet solution (Fuji Film Wako Pure Chemicals, Osaka, Japan). Bright-field images were captured using a BZ-X710 fluorescent imaging system (Keyence, Osaka, Japan). Quantification of colony staining was performed by measuring integrated density with ImageJ software (version 1.54d) [46].

### 4.8. Statistical Analysis

All statistical analyses were conducted using GraphPad Prism 9.0.0 (GraphPad Software, USA). Two-tailed unpaired Student’s *t*-tests were used for comparisons between two groups. For comparisons involving more than two groups, one-way ANOVA followed by Tukey’s post hoc test was applied. Two-way ANOVA with Tukey’s post hoc test was used for analyses involving two variables. A *p*-value less than 0.05 was considered statistically significant. Error bars in graphs represent the standard error of the mean (SEM).

## 5. Conclusions

In conclusion, this study reveals a novel interplay between KYNU and HDAC6 in regulating complement gene expression and glioblastoma cell viability. KYNU inhibition downregulates C3, C3AR1, and C5AR1, and induces cytotoxicity in part through upregulation of the cellular stress gene DDIT3. Conversely, while HDAC6 inhibition effectively suppresses GBM cell viability, it paradoxically upregulates KYNU, C3AR1, and C5AR1, potentially undermining its therapeutic efficacy. Notably, BET inhibition suppresses KYNU expression while restoring HDAC6 levels and exhibits synergistic cytotoxic effects when combined with tubastatin. Although we cannot rule out the possibility of off-target effects associated with tubastatin, our findings suggest that simultaneous targeting of HDAC6 and BET proteins (e.g., using tubastatin and apabetalone), may overcome complement-mediated resistance mechanisms within the GBM microenvironment. Further in vivo validation and mechanistic studies on KYNU-mediated complement regulation are needed. Nevertheless, our results provide compelling evidence for a novel therapeutic strategy in GBM, one that integrates epigenetic modulation with targeted regulation of the KYNU–complement axis to improve treatment efficiency within the immune microenvironment.

## Data Availability

The data analyzed during the current study are available from the corresponding author upon reasonable request.

## Supplementary Materials

The following supporting information can be downloaded at: https://www.mdpi.com/article/10.3390/epigenomes9030027/s1, Table S1: List of primers used in this study. Figure S1: NAD^+^ synthesis pathway and proposed KYNU-mediated regulation of the complement system in glioblastoma. Figure S2: KYNU expression in cancers and impact on progression-free survival. Figure S3: KYNU regulates inflammatory and hypoxic pathways. Figure S4: BET inhibitor apabetalone regulates KYNU and complement gene expression. Figure S5: HDAC6 is ubiquitously expressed in GBM cell lines. Figure S6: Tubastatin is less effective against high complement-expressing. Figure S7: KYNU inhibition attenuates HDAC6-mediated dysregulation of complement components. Figure S8: KYNU regulates HDAC6 expression. Figure S9: Inhibition of KYNU-HDAC6 mediated complement activation reduces GBM cell viability.
