# Supplementary material for: Targeting the Kynureninase–HDAC6–Complement Axis as a Novel Therapeutic Strategy in Glioblastoma
Source: Epigenomes. 2025 Jul 28;9(3):27. doi: 10.3390/epigenomes9030027 (PMC12372027; doi:10.3390/epigenomes9030027)
Supplement: Supplementary file 1 [file epigenomes-09-00027-s001.zip › epigenomes-3681480-supplementary.pdf]

Targeting the Kynureninase–HDAC6–Complement Axis as a Novel Therapeutic Strategy in Glioblastoma

Arif Ul Hasan *, Sachiko Sato, Mami Obara, Yukiko Kondo and Eiichi Taira

Department of Pharmacology, School of Medicine, Iwate Medical University, Yahaba 028-3694, Japan; sachikos@iwate-med.ac.jp (S.S.); mami@iwate-med.ac.jp (M.O.); ykondo@iwate-med.ac.jp (Y.K.); etaira@iwate-med.ac.jp (E.T.)

* Correspondence: hasan@iwate-med.ac.jp

Supplementary materials

Supplementary Table S1:

List of primers used in this study:

**Gene name**	**Forward primer**	**Reverse primer**
KYNU	GGAGGAATTGCTGGTGCCTT	AAACCATCCCACTAATGCAGG
C3	GGACTTGAAAGAGCCACCGA	CACAGCCAGAATCTCCCACG
C3AR1	GAAGATGCAGCGGACAGTGA	GGGGATGAGCTTGCATAGGA
C5AR1	GCTGACCATACCCTCCTTCC	CACGCCACACAACACCTTTG
IL6	AACCTGAACCTTCCAAAGATGG	TCTGGCTTGTTCCTCACTACT
IL10RB	AGCAAACAACCCATGACGAAAC	CAGACCATGAAGACCGAGGC
MAP3K8	ACTGTGGAGGATTTGCTTGCT	TGGGGAGTGATGACCATGTTT
CD55	AGAGTTCTGCAATCGTAGCTG	GGACGGCACTCATATTCCACA
VEGFA	CTTGCCTTGCTGCTCTACCT	GCTGCGCTGATAGACATCCA
HDAC6	CTAGATCGCTGCGTGTCCTT	TTCTAGGCTGTGAACCAACAT
DDIT3	AGAACCAGGAAACGGAAACAGA	TCTCCTTCATGCGCTGCTTT
B2M	TGCTGTCTCCATGTTTGATGTATCT	TCTCTGCTCCCCACCTCTAAGT
RPL13A *	CCTGGAGGAGAAGAGGAAAGAGA	TTGAGGACCTCTGTGTATTTGTCAA

(*For Figures 2E–H, Supplementary Figure S5C)

Supplementary Figures:

Supplementary Figure S1

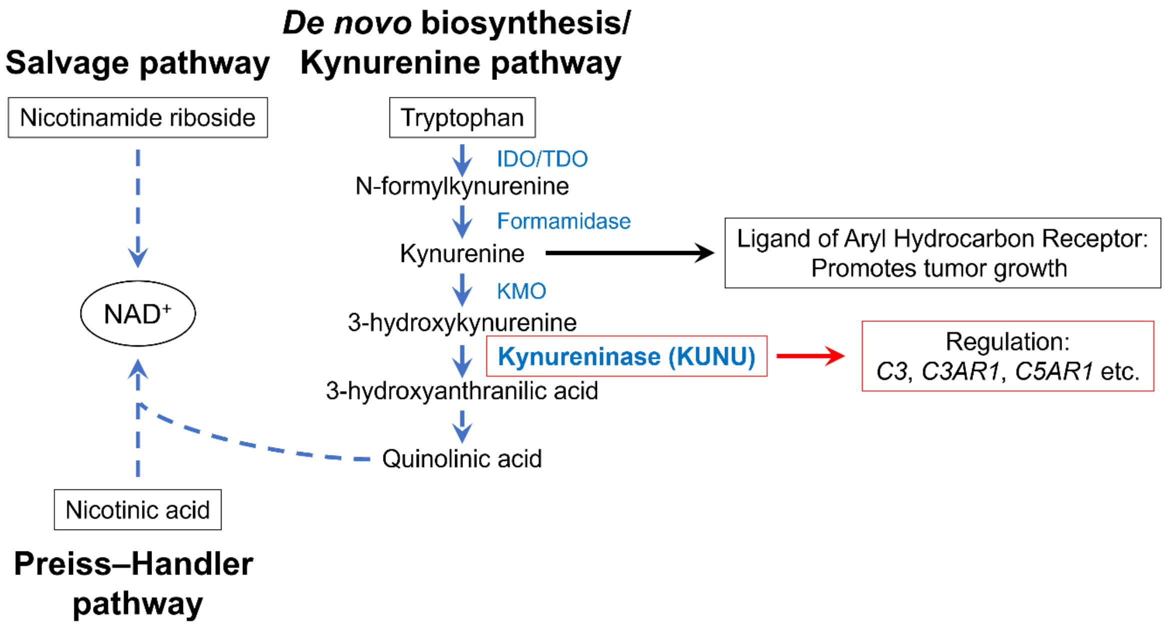

NAD^+ synthesis pathway and proposed KYNU-mediated regulation of the complement system in glioblastoma. NAD^+ is synthesized via tryptophan (Kynurenine pathway, also known as *De novo* biosynthesis pathway), nicotinic acid (Preiss–Handler pathway), and nicotinamide riboside (salvage pathway). KYNU is proposed as a novel regulator of selected complement genes (*C3*, *C3AR1*, *C5AR1*). Relevant molecules and pathways are shown; omitted parts are indicated by dotted lines. Enzymes are labeled in blue.

Supplementary Figure S2

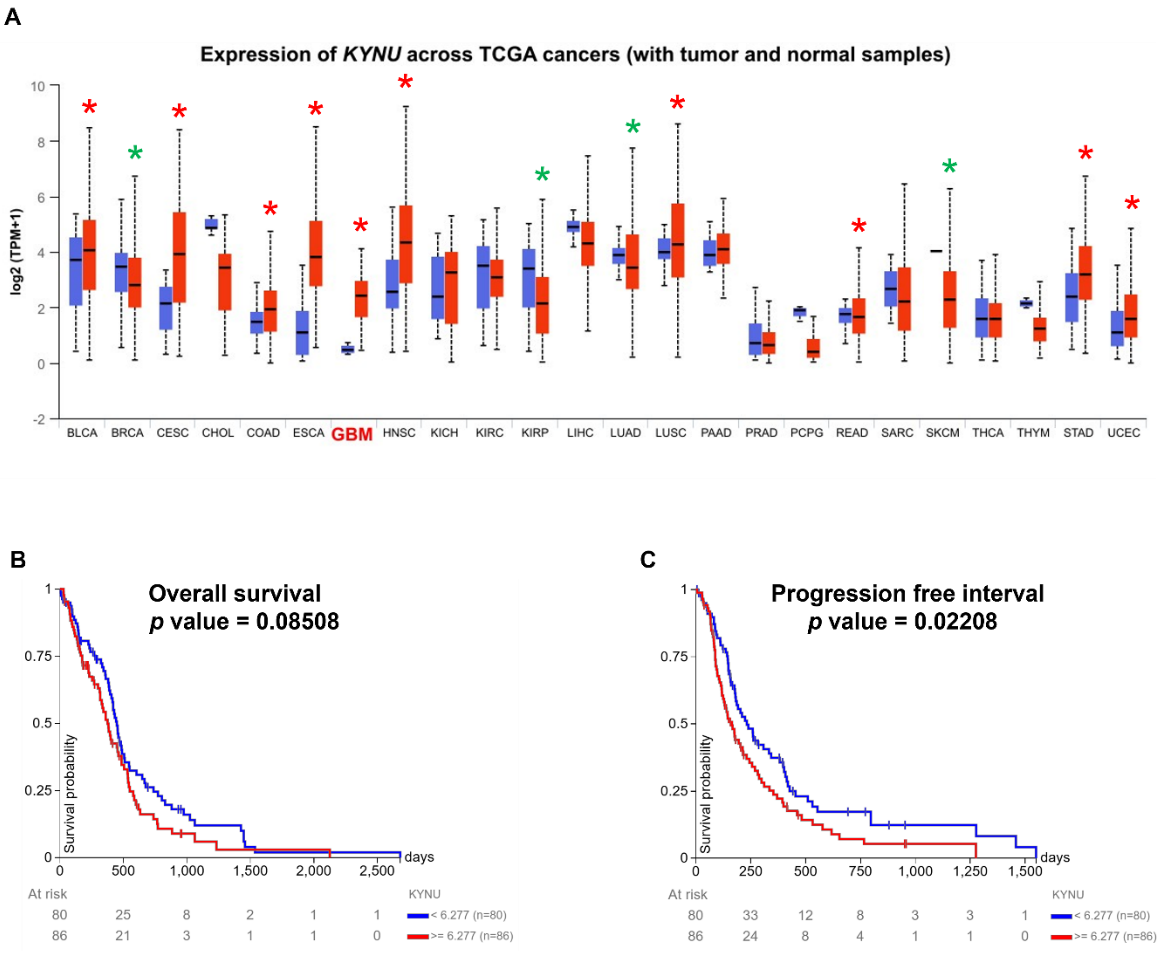

KYNU expression in cancers and impact on progression-free survival. (**A**) mRNA expression of *KYNU* in 24 tumor types versus corresponding normal tissues from TCGA, analyzed via UALCAN. Significant upregulation and downregulation are indicated by red and green asterisks, respectively (* $p < 0.05$; Student's *t*-test). (**B,C**) Kaplan–Meier survival curves for overall and progression-free survival in GBM patients with high (N = 86) versus low (N = 80) KYNU expression (above 3rd quartile). Curves compared using log-rank test via UCSC Xena. Cancer type abbreviations are as below:

BLCA: Bladder urothelial carcinoma, BRCA: Breast invasive carcinoma, CESC: Cervical squamous cell carcinoma, CHOL: Cholangiocarcinoma, COAD: Colon adenocarcinoma, ESCA: Esophageal carcinoma, GBM: Glioblastoma multiforme, HNSC: Head and Neck squamous cell carcinoma, KICH: Kidney Chromophobe, KIRC: Kidney renal clear cell carcinoma, KIRP: Kidney renal papillary cell carcinoma, LIHC: Liver hepatocellular carcinoma, LUAD: Lung adenocarcinoma, LUSC: Lung squamous cell carcinoma, PAAD:

Pancreatic carcinoma, PRAD: Prostate adenocarcinoma, PCPG: READ: Rectum adenocarcinoma, SARC: Sarcoma, SKCM: Skin cutaneous melanoma, THCA: Thyroid carcinoma, THYM: Thymoma, STAD: Stomach adenocarcinoma, and UCEC: Uterine Corpus Endometrial Carcinoma.

Supplementary Figure S3

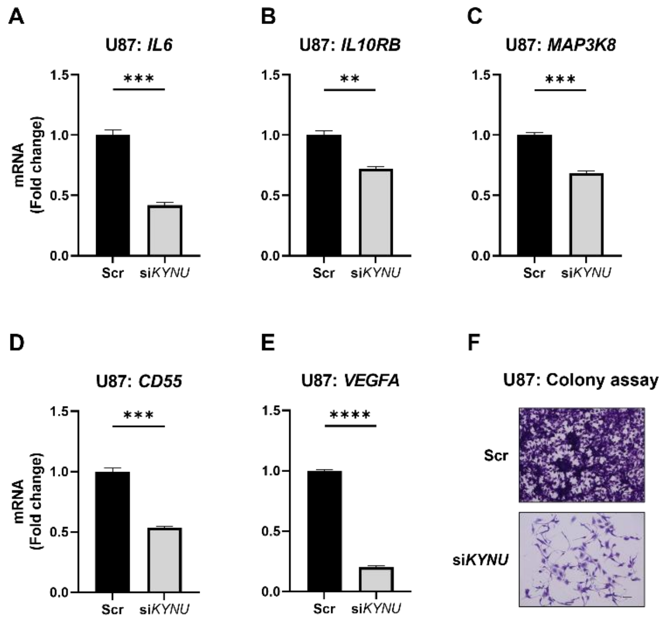

KYNU regulates inflammatory and hypoxic pathways. U87 glioblastoma cells were transfected with scrambled control siRNA (Scr) or *KYNU*-specific siRNA (si*KYNU*). (**A–E**) mRNA expression of target genes was quantified by qRT-PCR four days post-transfection (N = 3). (**F**) Colony formation was assessed by crystal violet staining eight days post-transfection. Statistical analysis: Two-tailed unpaired Student's *t*-test was applied to panels (**A–E**). *** $p < 0.001$; **** $p < 0.0001$. Bars represent mean + SEM. (**F**) Scalebar = 100 µm.

Supplementary Figure S4

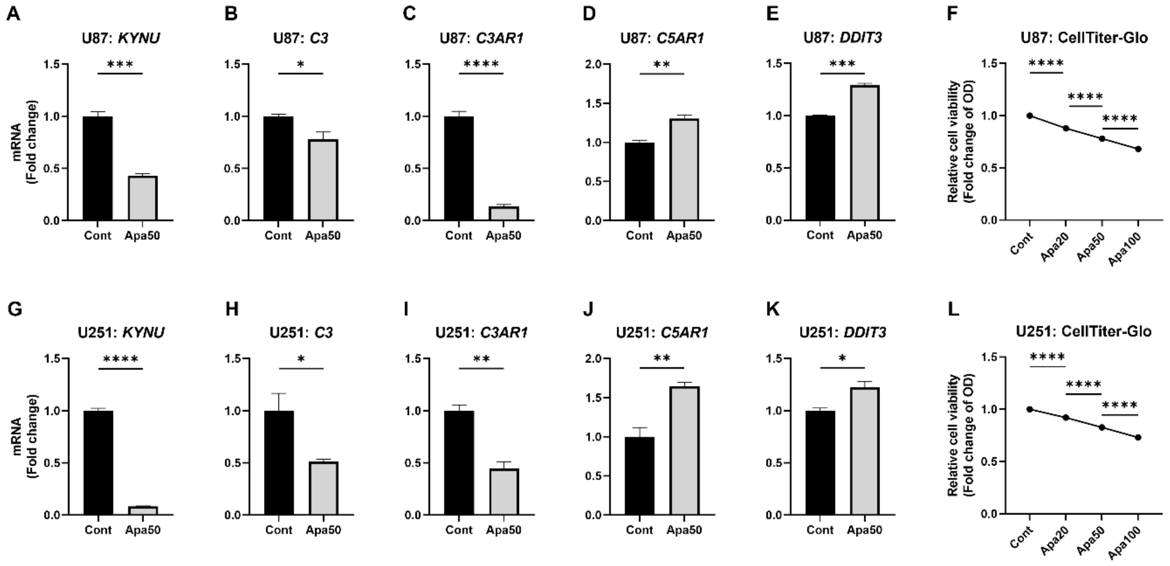

BET inhibitor apabetalone regulates KYNU and complement gene expression. U87 and U251 glioblastoma cells were treated with DMSO (Cont) or apabetalone (20, 50, or 100 μM). (**A–E,G–K**) mRNA expression of target genes was measured by qRT-PCR after 24 h of apabetalone treatment (N = 3). (**E,J**) Cell viability was measured using the CellTiter-Glo assay after 48 h of intervention (N = 4). Statistical analysis: Two-tailed unpaired Student's *t*-test was used for panels (**A–E,G–K**); two-way ANOVA with Tukey's post hoc test was applied for panels (**F,L**). * $p < 0.05$; ** $p < 0.01$; *** $p < 0.001$; **** $p < 0.0001$. Bars represent mean + SEM.

Supplementary Figure S5

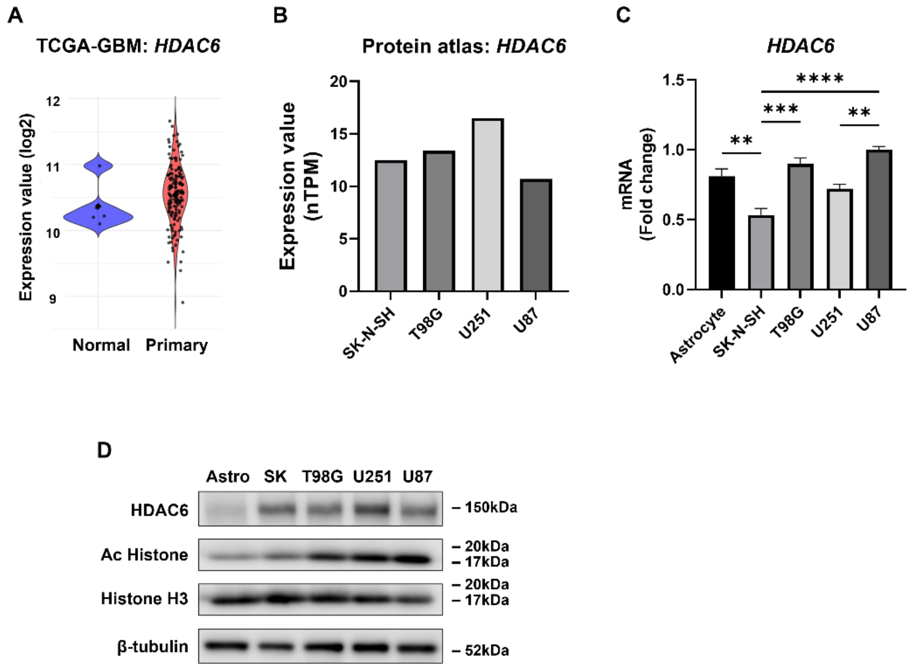

HDAC6 is ubiquitously expressed in GBM cell lines. (**A**) *HDAC6* mRNA expression was compared between normal brain tissue (Normal, N = 5) and primary GBM tissue (Primary, N = 154) using TCGA-GBM data accessed via the GDC portal. Differences in mean expression were estimated using nonparametric bootstrap resampling (10,000 iterations). Each dot represents an individual sample; large central points indicate group means. Bootstrapped 95% confidence intervals (CIs) for the mean difference are reported in the main text. Statistical significance was inferred if the 95% CI did not include zero. (**B**) *HDAC6* expression levels (normalized transcripts per million, nTPM) across various GBM cell lines, obtained from the Human Protein Atlas and GTEx. (**C**) Basal mRNA expression in selected cell lines was measured by qRT-PCR (N = 3), presented as fold change relative to U87. (**D**) Protein levels were assessed by immunoblotting, with β-tubulin used as a loading control. Statistical analysis: One-way ANOVA with Tukey's post hoc test was used for panel (**C**). ** $p < 0.01$; *** $p < 0.001$; **** $p < 0.0001$. Bars represent mean + SEM.

Supplementary Figure S6

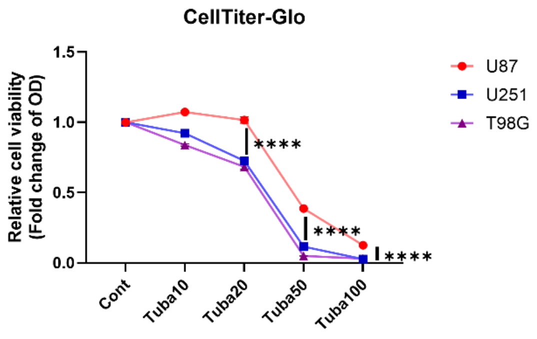

Tubastatin is less effective against high complement-expressing U87 cells. U87, U251, and T98G glioblastoma cells were treated with DMSO (Cont) or tubastatin (10, 20, 50, or 100 μM). Cell viability was measured using the CellTiter-Glo assay after 48 h of intervention (N = 4). Only relevant comparisons are shown. Statistical analysis: Two-way ANOVA with Tukey's post hoc test was applied. **** $p < 0.0001$. Bars represent mean + SEM.

Supplementary Figure S7

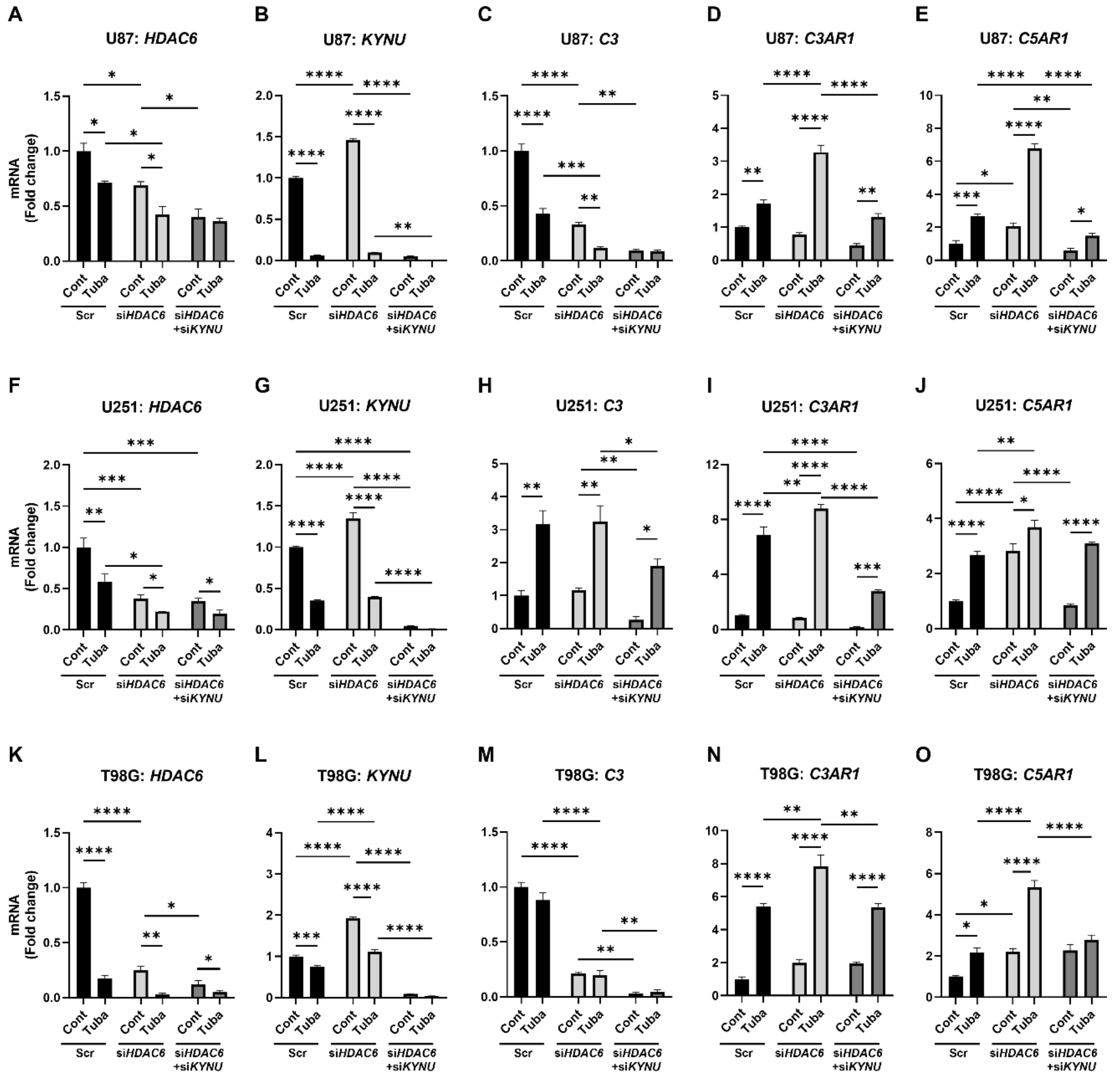

KYNU inhibition attenuates HDAC6-mediated dysregulation of complement components. U87, U251, and T98G cells were transfected with scrambled control siRNA (Scr), *HDAC6* specific siRNA (si*HDAC6*), or combined *HDAC6* and *KYNU* siRNAs (si*HDAC6*+si*KYNU*). Three days post-transfection, cells were treated with DMSO (Cont) or tubastatin (50 µM; Tuba). mRNA expression of target genes was assessed by qRT-PCR 24 h after tubastatin treatment. Statistical analysis: Two-way ANOVA with Tukey's post hoc test was applied to all panels. * $p < 0.05$; ** $p < 0.01$; *** $p < 0.001$; **** $p < 0.0001$. Bars represent mean + SEM.

Supplementary Figure S8

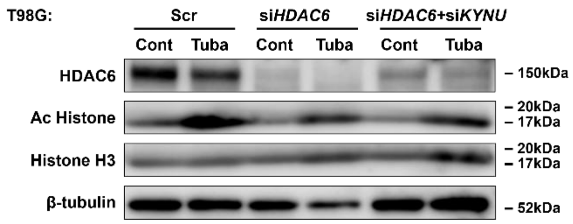

KYNU regulates HDAC6 expression. T98G cells were transfected with scrambled control siRNA (Scr), *HDAC6* specific siRNA (si*HDAC6*), or combined *HDAC6* and *KYNU* siRNAs (si*HDAC6*+si*KYNU*). Three days post-transfection, cells were treated with DMSO (Cont) or tubastatin (50 μM; Tuba). Protein levels were assessed by immunoblotting, with β-tubulin used as a loading control.

Supplementary Figure S9

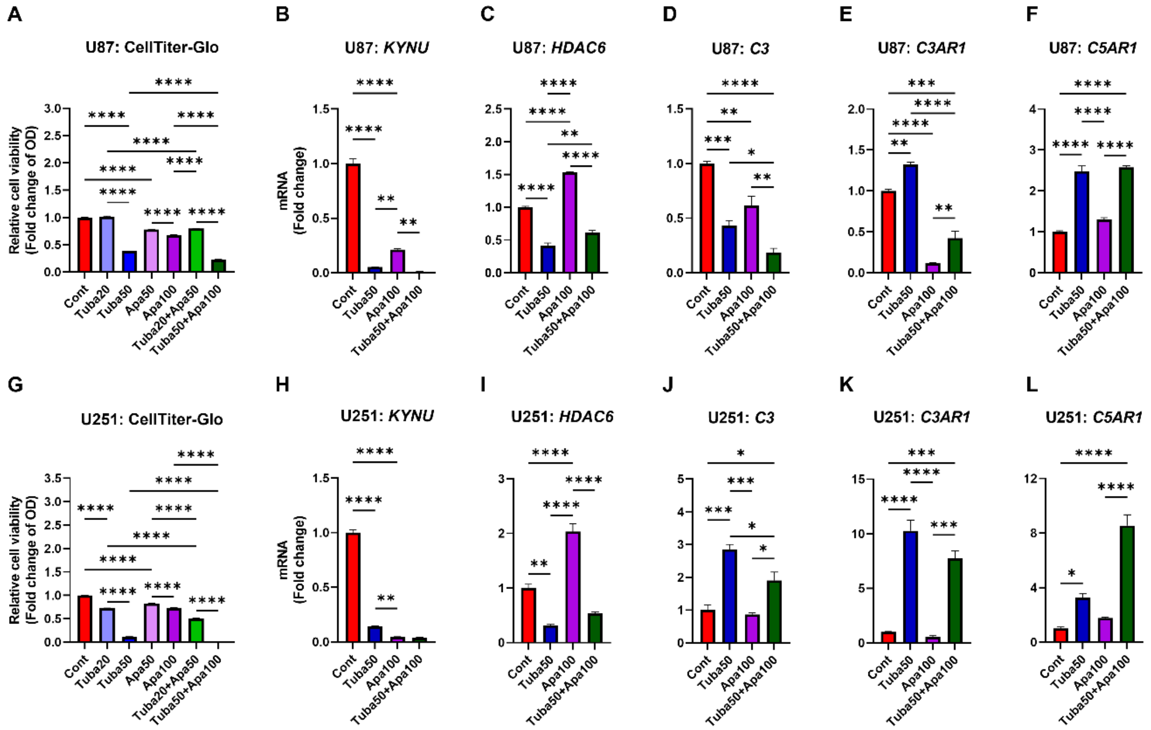

Inhibition of KYNU-HDAC6 mediated complement activation reduces GBM cell viability. U87 and U251 glioblastoma cells were treated with DMSO (Cont), tubastatin (20 or 50 μM), apabetalone (50 or 100 μM), or their combinations for the indicated durations. (**A,G**) Cell viability was assessed using the CellTiter-Glo assay after 48 h (N = 4). (**B**–**F, H**–**L**) mRNA expression of target genes was measured by qRT-PCR after 24 h of treatment (N = 3). Statistical analysis: One-way ANOVA with Tukey's post hoc test was used for all panels. * *p* < 0.05; ** *p* < 0.01; *** *p* < 0.001; **** *p* < 0.0001. Bars represent mean + SEM.
